# Theoretical Investigation of the Phenomenon of Space Charge Breakdown in Electromembrane Systems

**DOI:** 10.3390/membranes12111047

**Published:** 2022-10-26

**Authors:** Anna Kovalenko, Natalia Chubyr, Aminat Uzdenova, Makhamet Urtenov

**Affiliations:** 1 Department of Data Analysis and Artificial Intelligence, Kuban State University, Krasnodar 350040, Russia; 2 Department of Applied Mathematics, Kuban State University, Krasnodar 350040, Russia; 3 Department of Computer Science and Computational Mathematics, Umar Aliev Karachai-Cherkess State University, Karachaevsk 369202, Russia

**Keywords:** breakdown, space charge, desalination, membrane, electromembrane system

## Abstract

At present, it is customary to consider the overlimit operating modes of electromembrane systems to be effective, and electroconvection as the main mechanism of overlimiting transfer. The breakdown of the space charge is a negative, “destructive” phenomenon, since after the breakdown the size and number of electroconvective vortices are significantly reduced, which leads to a decrease in mass transfer. Therefore, electromembrane desalination processes must be carried out before space charge breakdown occurs. Thus, the actual problem arises of determining at which potential jumps a breakdown of the space charge occurs at a given concentration of the solution. Electromembrane systems are used for desalination at electrolyte solution concentrations ranging from 1 to 100 mol/m${}^{3}$. In a theoretical study of increasing the efficiency of the desalination process, mathematical modeling is used in the form of a boundary value problem for the system of Nernst–Planck and Poisson (NPP) equations, which refers to “hard” problems that are difficult to solve numerically. This is caused by the appearance of a small parameter at the derivative in the Poisson equation in a dimensionless form, and, correspondingly, a boundary layer at ion-exchange membranes, where concentrations and other characteristics of the desalination process change exponentially. It is for this reason that the numerical study of the boundary value problem is currently obtained for initial concentrations of the order of 0.01 mol/m${}^{3}$. The paper proposes a new numerical–analytical method for solving boundary value problems for the system of Nernst–Planck and Poisson equations for real initial concentrations, using which the phenomenon of space charge breakdown (SCB) in the cross section of the desalination channel in potentiostatic and potentiodynamic modes is studied. The main regularities of the appearance and interaction of charge waves, up to their destruction (breakdown), are established. A simple formula is proposed for engineering calculations of the potential jump depending on the concentration of the solution, at which the breakdown of the space charge begins.

## 1. Introduction

In works [1,2,3,4,5,6,7], it is shown that the use of overlimiting currents in electrodialyzers is effective. At overlimiting currents, the structure of the region of the space charge region becomes more complicated. The structure of the diffusion layer in the stationary case was first studied in [8] and later in the works of many authors [9,10,11,12]. Non-stationary problems were studied in [13,14,15,16]. In all these works, the numerical solution was obtained at initial concentrations much lower than in real electrodialyzers. This is due to the fact that the boundary value problems of mathematical models are “hard” problems, and the “hardness” increases with increasing initial electrolyte concentration. The reason is the appearance of a small parameter at the derivative in the Poisson equation, when passing to a dimensionless form using characteristic parameters, i.e., boundary value problems become singularly perturbed, which means the appearance of narrow boundary layers, where the desired functions of concentrations, electric field strength, etc., change exponentially. Moreover, the greater the initial concentration, the smaller the small parameter, and the more “rigid” the boundary value problem becomes and the more difficult it is to solve it numerically. Therefore, the main numerical results were obtained at initial concentrations of the order of 0.01 mol/m${}^{3}$, while the real initial concentration is of the order of 10 mol/m${}^{3}$ and more.

This paper proposes a new numerical–analytical method for solving boundary value problems for the system of Nernst–Planck and Poisson equations, which is a generalization of both works [17,18] and models [10,19,20,21,22]. This new method made it possible to study the non-stationary phenomenon of space charge breakdown in the desalination channel cross-section at real initial concentrations used in electrodialysis desalination apparatuses and to establish under these conditions the main patterns of interaction of charge waves, up to their destruction (breakdown) in potentiostatic and potentiodynamic modes. In [22], we applied the considered method to study breakdown at high concentrations in the galvanostatic mode. The essence of the work lies in the theoretical study of a new phenomenon—the breakdown of a space charge. It is for this reason that all equations and boundary conditions, as well as mathematical transformations, are displayed in the appendix.

## 2. Formation and Properties of a Quasi-Equilibrium Layer (QEL)

**Figure 1:**
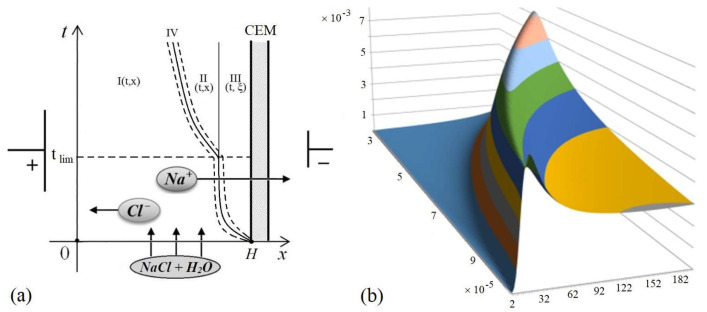
Formation and structure of the space charge region at cation-exchange membrane (CEM) (**a**) diffusion layer diagram (not to scale): I(t,x)—the region of electrical neutrality (REN), II(t,x)—extended space charge region (SCR), III(t,ξ)—QEL, IV(t,x)—intermediate region, and (**b**)—graph of the function $\rho \left(t,x\right)=F(C_{1}\left(t,x\right)-C_{2}\left(t,x\right))$.

In [14], the process of the appearance of a space charge region in the potentiodynamic mode in the diffusion layer near the cation-exchange membrane (CEM) was considered, assuming that the potential jump increases linearly with time, starting from zero. As can be seen from Figure 1a, most of the diffusion layer is occupied by the region of electrical neutrality (REN)—I(t,x), at which $C_{1}=C_{2}$ or ε=0. In the region I(t,x), the electromigration and diffusion fluxes are exactly equal, therefore the concentrations of ions of opposite signs at each point at any time are equal, and therefore the conditions of local electroneutrality are observed. It follows from Figure 1 that the formation of a quasi-equilibrium region of the space charge (III(t,ξ)) adjacent to the ion-exchange membrane begins at the initial moment of time, its thickness increases nonlinearly with time (Figure 1b) and at some point practically stops changing (Figure 1a). The transfer of salt ions in this region is practically independent of time and, accordingly, of the potential jump and, consequently, of the current. Therefore, this region of space charge (III(t,ξ)) is called a quasi-equilibrium region or layer (QEL) [23]. The reason for the formation of QEL is that, at short times, the migration flow near the membrane in the (III(t,ξ)) region is not much greater than the diffusion flow, and the flows themselves are directed oppositely, as a result of which counterions accumulate near the membrane. As time increases, the predominance of the electromigration flow over the diffusion one increases. This leads to the fact that with a further increase in time, at some $t_{lim}$, i.e., when the potential jump becomes large enough and the current corresponding to it becomes greater than the limiting diffusion current, then counterions begin to accumulate at the boundary between the electroneutrality region and the QEL, since diffusion does not have time to blur their accumulation (because the diffusion flux is less than the electromigration flux). Thus, an extended region of space charge (II(t,x)) appears. The thickness of the QEL is practically independent of time, i.e., the quasi-equilibrium layer is simultaneously quasi-stationary (Figure 1). In the cross section of the desalination channel formed by anion-exchange and cation-exchange membranes, quasi-equilibrium layers appear at each of the membranes, and are also quasi-stationary [23,24] and Section 4.

## 3. Formation of an Extended Space Charge Region and Local Extrema of the Space Charge

Numerical experiments [25] and analytical calculations show that, at a fixed value of the exchange capacity of an ion-exchange membrane, a local extremum of the space charge appears at a certain potential jump corresponding to the overlimiting current density. If a potential jump is fixed, then at a certain sufficiently large value of the exchange capacity of the membrane, the local extrema disappear. Based on this, it was suggested in [26] that the reason for the occurrence of local extrema is the limited exchange capacity of membranes.

**Figure 2:**
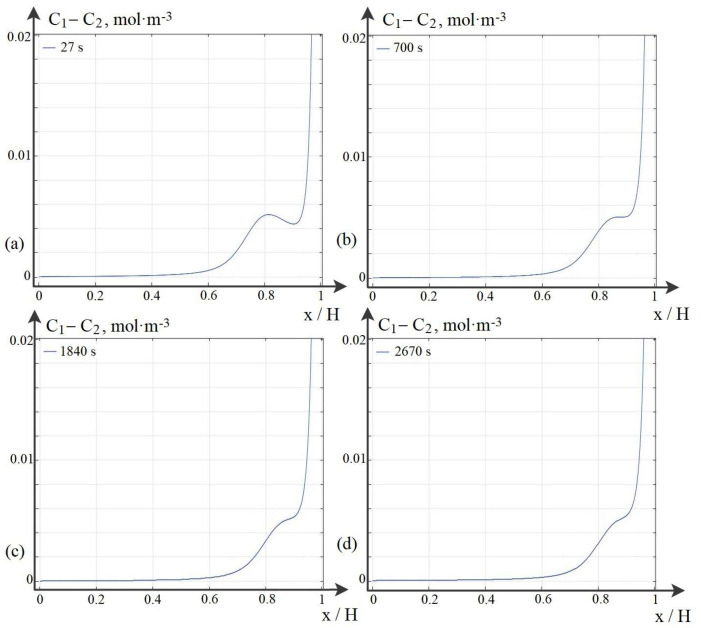
Dependence of the local maximum on the exchange capacity of the cation-exchange membrane (NaCl, $\Delta_{r}\varphi =0.5$) (**a**) $C_{1m}=14$, (**b**) $C_{1m}=350$, (**c**) $C_{1m}=920$, (**d**) $C_{1m}=1335$.

Let us consider the dependence of the local maximum on the $C_{1m}$ parameter, which characterizes the exchange capacity of the membrane. The point of the local maximum shifts to the right, but the value practically does not change (Figure 2). At the same time, the local minimum gradually fills up with an increase (the value of the local minimum increases). Thus, we can conclude that the local maximum of the space charge appears due to the limited exchange capacity of the membrane at a given potential jump, i.e., the local maximum of the space charge appears due to the presence of a local minimum of the space charge at the surface of the cation exchange membrane. In the potentiodynamic regime, when the potential jump increases linearly with a certain sweep rate, as shown in [27], the existence of local extrema of the plot of current density vs. potential jump (time) is related to the potential sweep rate. If the sweep rate is low, then there are no local extrema. Both in the first and in the second case, everything is connected with the ratio of diffusion and electromigration. At large values of the exchange capacity, the concentration gradient increases and diffusion begins to “prevail” over electromigration. Similarly, with a decrease in the sweep rate, the electromigration flow decreases and diffusion also “prevails” over electromigration, and thus, if the potential jump is slowly increased, local maxima and minima are not formed; however, the extended space charge region will increase.

Thus, the prevalence of diffusion over electromigration does not allow the formation of local extrema of the space charge, and vice versa, the prevalence of electromigration over diffusion leads to the formation of local extrema. If the potential jump is taken constant and small enough, then although the electromigration flux will slightly exceed the diffusion flux, they will be approximately equal and there will be no accumulation of ions at the boundary of the electroneutrality region and QEL. At small potential jumps, the QEL smoothly passes into the region of electrical neutrality, there are no boundaries between them and, accordingly, there is no extended region of the space charge. If the potential jump is taken as constant and large enough, then the migration flow will be much larger than the diffusion flow at the boundary between the electroneutrality and QEL regions, which will lead to the accumulation of ions in this region and the formation of an extended SCR.

## 4. Analytical Solution of the Boundary Value Problem in Quasi-Equilibrium Layers in the Section of the Desalination Channel

Let us first find the solution on the segment $\left[0,x_{1}\right]$, that is, in the quasi-equilibrium boundary layer near the anion-exchange membrane. Let us put (1) and (2), then we substitute in the dimensionless Nernst–Planck and Poisson equations (see Appendix A.1), then after a series of transformations near the anion-exchange membrane, in the first approximation, we obtain (3) with boundary conditions (4), and also E(t,∞,ε)=0—the condition of merging with the solution inside the channel section, from which follows $j_{1}\left(t\right)\equiv 0$.

(1) $$\xi =\frac{x}{\sqrt{\varepsilon }},E\left(t,x,\varepsilon \right)=\frac{1}{\sqrt{\varepsilon }}\tilde{E}\left(t,\xi ,\varepsilon \right),C_{1}\left(t,x,\varepsilon \right)=C_{1}\left(t,\xi ,\varepsilon \right)$$

(2) $$C_{2}\left(t,x,\varepsilon \right)=C_{2}\left(t,\xi ,\varepsilon \right),j_{1}\left(t,x,\varepsilon \right)=j_{1}\left(t,\xi ,\varepsilon \right),j_{2}\left(t,x,\varepsilon \right)=j_{2}\left(t,\xi ,\varepsilon \right)$$

(3) $$j_{i}\left(t,\xi ,\varepsilon \right)=j_{i}\left(t\right),\frac{\partial C_{i}\left(t,\xi ,\varepsilon \right)}{\partial \xi }=z_{i}C_{i}\tilde{E}\left(t,\xi ,\varepsilon \right),i=1,2,\frac{\partial \tilde{E}\left(t,\xi ,\varepsilon \right)}{\partial \xi }=C_{1}-C_{2}.$$

(4) $$j_{1}\left(t,0,\varepsilon \right)=\left(C_{1}\tilde{E}-\frac{\partial C_{1}}{\partial \xi }\right)\left(t,0\right)=0,C_{2}\left(t,0,\varepsilon \right)=C_{2a}=1,\varphi \left(t,0\right)=dt.$$

The system of Equations (A17) and (A18) (see Appendix A.1) has the first integral $C_{1}+C_{2}=\frac{1}{2}{\tilde{E}}^{2}-\alpha$, where $\alpha =-C_{1}\left(t,x_{1},\varepsilon \right)-C_{2}\left(t,x_{1},\varepsilon \right)<0$. Using which, one can obtain an equation for the electric field strength that does not contain concentrations (5) [22]. Integrating which, after a series of transformations, we obtain (6). The condition of merging with the solution in the REN: $\tilde{E}\to 0$ is obviously satisfied; moreover, it is possible to define the left boundary of the REN in the form $x_{1}=k\sqrt{\varepsilon }\left|ln\varepsilon \right|$, where k>0 is an arbitrary constant.

(5) $$\frac{\partial^{2}\tilde{E}}{\partial x^{2}}=\frac{1}{2}{\tilde{E}}^{3}-\alpha \tilde{E}.$$

(6) $$\tilde{E}=\frac{4\sqrt{\beta }e^{-\sqrt{-\alpha }\xi }}{1-\beta e^{-\sqrt{-4\alpha }\xi }}\sqrt{-\alpha }.$$

Thus, we obtain solution (7):

(7) $$E\left(x,\varepsilon \right)=\frac{1}{\sqrt{\varepsilon }}\frac{4\sqrt{\beta }e^{-\sqrt{-\alpha }\frac{x}{\sqrt{\varepsilon }}}}{1-\beta e^{-\sqrt{-4\alpha }\frac{x}{\sqrt{\varepsilon }}}}\sqrt{-\alpha },$$

where β—some positive number, which is determined from the boundary condition. Knowing E(x,ε), and using the ratios $C_{1}+C_{2}=\frac{1}{2}{\tilde{E}}^{2}-\alpha$ and $\frac{\partial \tilde{E}}{\partial \xi }=C_{1}-C_{2}$, it is easy to calculate $C_{1}$ and $C_{2}$. Similarly, the solution is calculated on the segment $\left[x_{1},1\right]$, that is, in the quasi-equilibrium layer near the cation-exchange membrane with the necessary changes, namely, the replacement has the form: $\xi =\frac{x-1}{\sqrt{\varepsilon }}$, $E=\frac{1}{\sqrt{\varepsilon }}\tilde{E}\left(\xi ,\varepsilon \right)$, ξ→−∞, at ε→+0, which leads to the solution (8), where $\alpha \approx -C_{1}\left(t,x_{2},\varepsilon \right)-C_{2}\left(t,x_{2},\varepsilon \right)<0$.

(8) $$E\left(x,\varepsilon \right)=\frac{1}{\sqrt{\varepsilon }}\frac{4\sqrt{\beta }e^{-\sqrt{-\alpha }\frac{x-1}{\sqrt{\varepsilon }}}}{1-\beta e^{-\sqrt{-4\alpha }\frac{x-1}{\sqrt{\varepsilon }}}}\sqrt{-\alpha },$$

As can be seen from the solutions in quasi-equilibrium layers, in the first approximation they do not depend on time, that is, the quasi-equilibrium layer is also quasi-stationary.

## 5. Model Formulation wQEL (without Quasi-Equilibrium Boundary Layer)

As shown above, in the initial approximation, the value of the current density does not affect the distribution of the potential and concentrations of the quasi-equilibrium region of the space charge. This influence affects only in the next, first, approximation [14]. In this regard, in the initial approximation, this region can be ignored and a simplified model can be compiled, and the conditional boundary between the quasi-equilibrium region of the space charge and the extended region can be considered the point at which the concentration of counterions reaches its minimum value for ion-exchange membranes, and the space charge reaches a minimum at CEM and the maximum value of AEM. Since in the vicinity of these points the values of the concentration of counterions are much higher than the concentration of coions, then, accordingly, the space charge in these regions is determined by the concentration of counterions.

Thus, we can conclude that $\frac{\partial C_{1}}{\partial x}\approx \frac{\partial \rho }{\partial x}\approx 0$ at the border of the extended region and $\frac{\partial C_{2}}{\partial x}\approx \frac{\partial \rho }{\partial x}\approx 0$ the quasi-equilibrium region of the cation-exchange membrane and at the border of the expanded region and the quasi-equilibrium region of the anion-exchange membrane. Since the width of the quasi-equilibrium region is rather small, the following boundary conditions can be adopted to simplify the basic model (9):

(9) $$\frac{\partial C_{2}\left(t,0\right)}{\partial x}=0,\frac{\partial C_{1}\left(t,H\right)}{\partial x}=0.$$

Adding to these conditions the conditions of impermeability of coions, the same as in the basic problem, we obtain a new boundary value problem for the system of Nernst–Planck–Poisson equations, which determines the mathematical model for the transport of salt ions without a quasi-equilibrium layer (without quasi-equilibrium boundary layer (wQEL)). Thus, the model without a quasi-equilibrium layer is described by Equations (A1)–(A5) and boundary conditions (9), (A6)–(A14). Since this model differs from the basic model in the absence of a quasi-equilibrium space charge region, it can be called a model without a quasi-equilibrium layer. In a number of papers, the authors investigated this model of salt ion transport in a diffusion layer, and it was shown that it gives the distribution of concentration, potential, and space charge with good accuracy everywhere except, of course, the quasi-equilibrium region of the space charge.

As the calculations performed in this article show, the wQEL model allows one to numerically study the transfer phenomenon in the cross section of the desalination channel for an electrolyte solution with higher concentrations than the basic model, for example, for initial concentrations $C_{0}=10$ mol·m${}^{-3}$.

In [14], the quasi-uniform charge distribution (QCD) condition was proposed, which generalizes the electrical neutrality condition. As a result of using this condition instead of the Poisson equation, a finite equation or a differential equation of a lower order is obtained, and as a result, a solution is obtained outside the quasi-uniform layer. In this sense, the wQEL model and the QCD condition lead to the same results. The wQEL model is convenient for numerical solution, since only two boundary conditions change. The QCD condition was derived using the decomposition method of the system of Nernst–Planck and Poisson equations, and the model with the QCD condition is convenient for an asymptotic solution.

Above, the wQEL model was derived in dimensionless form. However, it is not difficult to formulate it in dimensional form, since the conditions (9) have the same form in dimensional form. In addition, it is easy to pass to the dimensional form in Formulas (7) and (8).

## 6. A New Numerical-Analytical Method for Solving Boundary Value Problems for the System of Nernst-Planck and Poisson Equations

### 6.1. Algorithm of the Numerical-Analytical Method of Solution

1. We numerically solve the boundary value problem (A15)–(A24) of the wQEL model, and find, among other things, $C_{1}\left(t,x_{2},\varepsilon \right),C_{2}\left(t,x_{1},\varepsilon \right)$;
2. We find the potential jump for the base model. For this, we use the ratio

$\varphi_{0}=\int_{0}^{1}E\left(x,\varepsilon \right)\;dx=\int_{0}^{x_{1}}E\left(x,\varepsilon \right)\;dx+\int_{x_{1}}^{x_{2}}E\left(x,\varepsilon \right)\;dx+\int_{x_{2}}^{1}E\left(x,\varepsilon \right)\;dx,$

$\varphi_{0}=\int_{0}^{1}E\left(x,\varepsilon \right)\;dx=-\int_{0}^{x_{1}}\frac{dC_{2}}{C_{2}}+\int_{x_{1}}^{x_{2}}E\left(x,\varepsilon \right)\;dx+\int_{x_{2}}^{1}\frac{dC_{1}}{C_{1}}=ln\frac{C_{2A}C_{1K}}{C_{2}\left(t,x_{1},\varepsilon \right)C_{1}\left(t,x_{2},\varepsilon \right)}+\int_{x_{1}}^{x_{2}}E\left(x,\varepsilon \right)\;dx.$

  Taking into account that $x_{1}\approx 0,x_{2}\approx 1$, we obtain

$\varphi_{0}\approx ln\frac{C_{2A}C_{1K}}{C_{2}\left(t,x_{1},\varepsilon \right)C_{1}\left(t,x_{2},\varepsilon \right)}+\int_{x_{1}}^{x_{2}}E\left(x,\varepsilon \right)\;dx,$

$\varphi_{0}=\varphi_{QEL}+\varphi_{wQEL}.$

  Here, the first term $\varphi_{QEL}$ is the potential jump in the quasi-equilibrium layers of the anion-exchange and cation-exchange membranes, and the second potential jump is $\varphi_{wQEL}$, calculated using the wQEL model. Let us estimate the potential jump $\varphi_{QEL}$, assuming that the minimum value of the concentration has decreased by 100 and $10^{5}$ times.
  Then, in the first case, we obtain $\frac{C_{2A}}{C_{2}\left(t,x_{1},\varepsilon \right)}=\frac{C_{1K}}{C_{1}\left(t,x_{2},\varepsilon \right)}=10^{2}$, and in the second $\frac{C_{2A}}{C_{2}\left(t,x_{1},\varepsilon \right)}=\frac{C_{1K}}{C_{1}\left(t,x_{2},\varepsilon \right)}=10^{5}$.
  Then, the dimensionless jumps will be $\varphi_{QEL}=ln\frac{C_{2A}C_{1K}}{C_{2}\left(t,x_{1},\varepsilon \right)C_{1}\left(t,x_{2},\varepsilon \right)}\approx 9.2$ and $\varphi_{0}=ln\frac{C_{2A}C_{1K}}{C_{2}\left(t,x_{1},\varepsilon \right)C_{1}\left(t,x_{2},\varepsilon \right)}\approx 23$.
  Taking into account that $\varphi_{0}=0.02566$ V, we obtain that in dimensional form the total potential jump in quasi-equilibrium layers is approximately equal to 0.24 V and 0.6 V.
  Taking into account the fact that the potential jump in the desalination chamber can reach 1 V–3 V, the potential jump in quasi-equilibrium layers can make a significant contribution with an increase in the degree of desalination.
3. We find an analytical solution in quasi-equilibrium layers using Formulas (7) and (8).
4. Using (1) and (3), we obtain the solution of the basic problem.

**Remark** **1.** *As $C_{0}$ increases, the asymptotic solution becomes more accurate ε, since the accuracy of analytical formulas decreases and, accordingly, increases, and the thickness of the quasi-equilibrium layer decreases (see Table A1). Thus, in contrast to the numerical solution of the boundary value problem of the base model, the accuracy of the proposed numerical-analytical method of the solution practically does not change with increasing $C_{0}$.*

### 6.2. Verification of Calculations

To verify the calculations, numerical experiments were carried out with grids with different numbers of elements 200,000, 330,000, and 400,000. The calculation results in the first two cases differed, although slightly. The results of calculations for grids of 400,000 and 330,000 coincided with the accuracy of the calculations. Therefore, calculations with a grid of 400,000 can be considered quite accurate.

### 6.3. Comparison of the Results of Calculations of the Base Model and the wQEL Model

**Figure 3:**
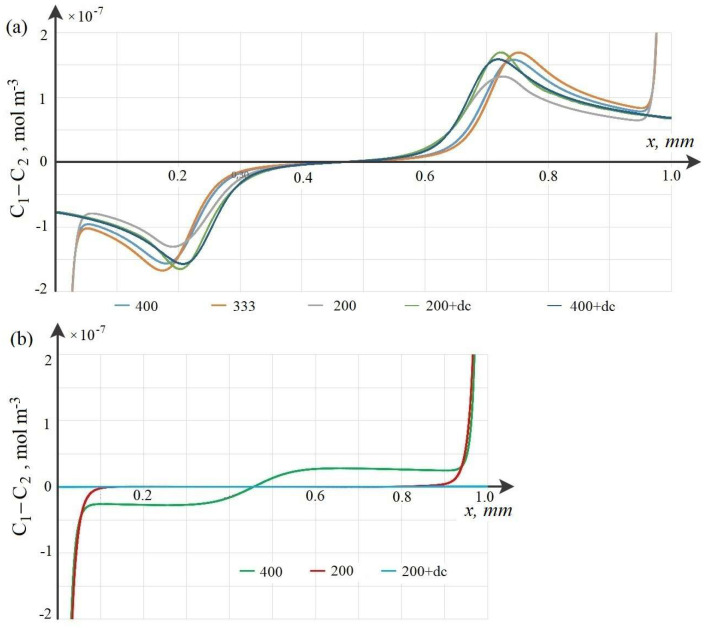
Calculations at different time intervals: before breakdown (**a**), on the eve of breakdown and after breakdown (**b**).

At low initial concentrations, the basic model and the wQEL model can be used for calculations simultaneously. Such a comparison is made below for $C_{0}=0.01$ mol·m${}^{-3}$ at the same potential jumps. As can be seen from Figure 3, the distribution of the space charge calculated by the wQEL model coincides quite accurately everywhere, except, of course, for the quasi-equilibrium region of the space charge. The exclusion of this region leads to some delay in the values of the space charge calculated by the simplified model in comparison with the base one. This delay depends on the potential jump in the quasi-equilibrium region of the space charge, which in turn depends on the initial concentration (A12) and (A13), for example, for the concentration $C_{0}=0.01$ mol·m${}^{-3}$ the delay is 15 s or, which is the same, 15 s ·0.005 V/s =0.075 V. If this shift is taken into account, the results differ by less than 1%. From Appendix A.2.1, Appendix A.2.2 and Appendix A.2.3 it follows that the wQEL model in combination with the analytical solution in the quasi-equilibrium region and taking into account the jump in this region, i.e., the proposed numerical–analytical method can be used to calculate the transfer of ions in the cross section of the desalting channel, including the phenomenon of space charge breakdown at the real initial solution concentrations used for desalting in electrodialysis apparatuses.

## 7. Patterns of Space Charge Breakdown at High Initial Concentrations

The concept of space charge breakdown and the main patterns of breakdown in non-stationary membrane systems at fixed potential jumps corresponding to the overlimiting current density were studied in [25] at small values of the initial concentration of the order of $C_{0}=0.01$ mol·m${}^{-3}$, which, as noted above, is due to the fact that boundary value problem (A15)–(A24) is a singularly perturbed problem due to the small parameter ε, and therefore ill-conditioned. Moreover, with an increase in $C_{0}$, the value ε decreases (see Table A1).

**Figure 4:**
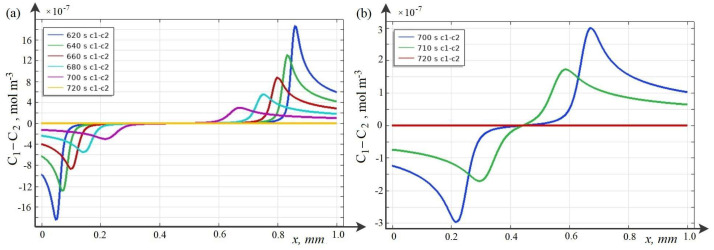
Breakdown phenomenon for a KCl solution with initial concentration $C_{0}=10$ mol·m${}^{-3}$. Space charge graphs normalized to Faraday numbers at different time intervals: (**a**) before breakdown, and (**b**) before and after breakdown.

**Figure 5:**
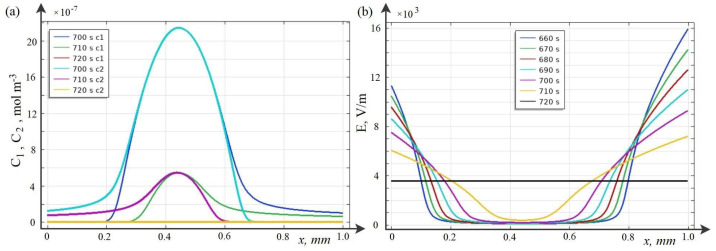
Graphs of concentrations (**a**) and intensity (**b**) at different points in time.

**Figure 6:**
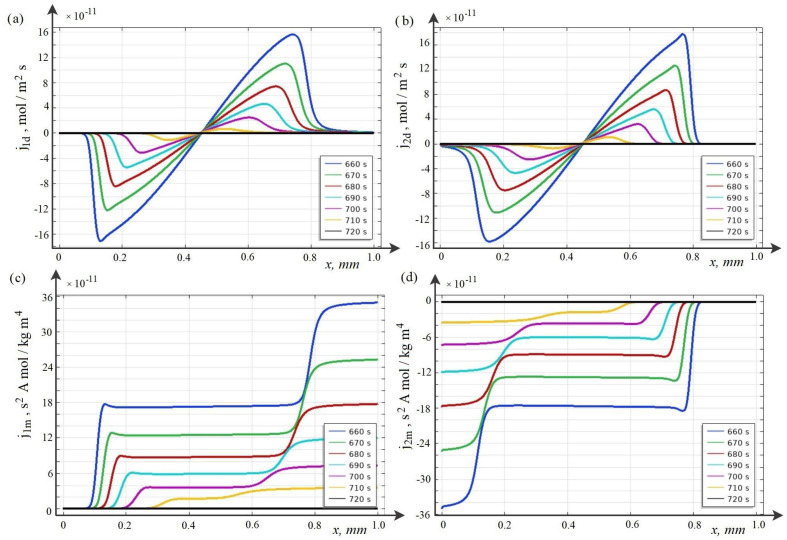
Graphs of diffusion (**a**,**b**) and migration (**c**,**d**) fluxes of K${}^{+}$ and Cl${}^{-}$ ions at different points in time.

Below, the breakdown phenomenon and its regularities for the potentiodynamic mode are studied using the wQEL model in the cross section of the desalination channel for $C_{0}=10$ mol·m${}^{-3}$. As time increases, two waves of a positive (for CEM) and a negative (for AEM) space charge are formed in the channel cross section, which move towards each other (Figure 4a and Figure 5). These waves are caused by the predominance of migration flows over diffusion ones (see Figure 6a–d).

In this case, the concentration of cations is higher than the concentration of anions in the area adjacent to the cation-exchange membrane and, accordingly, the concentration of anions is higher than the concentration of cations in the area adjacent to the anion-exchange membrane (Figure 5a). Accordingly, the space charge in the region adjacent to the cation exchange membrane is positive, and in the region adjacent to the anion exchange membrane it is negative. In the middle part of the channel, there is a region where the concentrations of cations and anions are equal with a high accuracy, and in this region the condition of local electrical neutrality is satisfied. In the areas of the space charge, the intensity is very high, the convexity and concavity of its graph at the extremum points of the space charge change (Figure 5b).

Calculations show that at first the space charge waves move with almost constant speed and do not interact. Over time, they approach each other and begin to attract each other, the speed of movement gradually increases. At some point in time (Figure 4b), the waves of negative and positive space charge come into contact and the discharge process begins, when the value of both negative and positive charges decreases rather quickly and over time, the space charge in the middle part of the channel practically disappears, that is, the breakdown process is completed (Figure 4b).

As can be seen from Figure 4, the breakdown occurs at 700–720 s. Since the sweep rate is d=0.005 V·s${}^{-1}$, we find that the breakdown occurs at approximately 3.5–3.6 V.

After the breakdown, the flows become almost equal to zero (Figure 6), and, accordingly, the current becomes equal to zero, equilibrium is established in the entire section of the channel, and the concentration and tension in the middle part of the channel become constant (Figure 5). The quasi-equilibrium layers of the membranes are retained [14].

## 8. Dependence of the Time (Potential Jump) of the Onset of Breakdown on the Initial Concentration of the Solution

**Figure 7:**
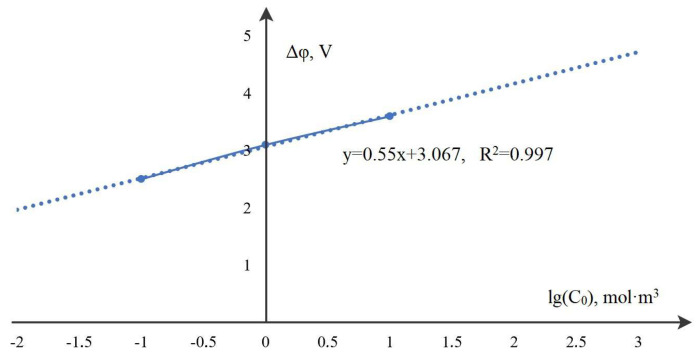
Dependence of the jump in the breakdown start potential $\varphi_{n}$ for a KCl solution on the logarithm of the initial concentration $C_{0}$ at a channel width of 1 mm.

The equation of the straight line in Figure 7 has the form

$$\varphi_{n}=alg\left(C_{0}\right)+b,$$

where a=0.54, b=3.07, $R^{2}=0.9973$. Assuming a=0.5, b=3, we obtain a simple approximate dependence for a preliminary estimate of the critical value of the potential jump at which the breakdown begins:

$$\varphi_{new}=\frac{1}{2}lg\left(C_{0}\right)+3.$$

It follows from Table 1 that this formula is quite accurate and can be used in engineering calculations to estimate the potential jump when the breakdown of the space charge begins for a solution with an initial concentration $C_{0}$.

## 9. Conclusions

This paper proposes a new mathematical model for the transfer of salt ions in the cross section of the desalination channel with the exception of quasi-equilibrium layers near ion-exchange membranes, called the wQEL model. Asymptotic solutions are found in quasi-equilibrium layers of ion-exchange membranes. Using a combination of the analytical solution and the numerical solution of the wQEL model, a numerical–analytical method for solving the basic model was developed, using which the breakdown phenomenon in the cross section of the desalination channel was theoretically studied at real concentrations of the initial electrolyte solution. The main patterns of breakdown are determined, which can be used to select effective technological parameters for the operation of an electrodialysis desalination apparatus:

(1) For the first time, a study of a quasi-equilibrium layer in a non-stationary case was carried out and it was shown that its thickness is practically independent of time, i.e., the quasi-equilibrium layer is also quasi-stationary.

(2) It is shown that at overlimiting current densities (potential jumps) in the potentiostatic mode, space charge waves arise at ion-exchange membranes with local extrema, which move towards each other and at the moment of meeting they are discharged, i.e., breakdown of the space charge occurs. It is shown for the first time that the cause of local extrema is the predominance of the electromigration flow over the diffusion one.

(3) For the first time for the potentiostatic mode, a simple and fairly accurate formula for engineering calculations has been derived, the formula for the dependence of the jump in the breakdown start potential for a KCl solution on the logarithm of the initial concentration $C_{0}$, which allows you to determine in advance the potential jump acceptable for desalination at a given concentration.

(4) It has been shown for the first time that in the potentiodynamic regime with a linear sweep rate of the potential, space charge waves with local extrema arise at sweep rates greater than a certain threshold value, which depends on the concentration of the solution.

(5) A new mathematical model of ion transport in the cross section of the desalination channel has been proposed, using which a hybrid numerical–analytical solution method has been developed. This method can be used to solve other problems of membrane electrochemistry, where the appearance of a quasi-equilibrium layer makes it difficult to use only numerical methods.

## Figures and Tables

**Table 1:** Breakdown start time $t_{n}$ (potential jump, $\varphi_{n}$) depending on the initial concentration of the KCl solution.

$C_{0},$ mol/m${}^{3}$	0.01	0.1	1	10	100
$t_{n}$	400	500	620	720	820
$\varphi_{n},V,numeric$	2	2.5	3.1	3.6	4.1
$\varphi_{new},V$	2	2.5	3	3.5	4

## Data Availability

Not applicable.

## Abbreviations

The following abbreviations are used in this manuscript:

NPP	Nernst-Planck and Poisson
SCB	Space charge breakdown
QEL	Quasi-equilibrium layer
CEM	Cation-exchange membrane
AEM	Anion exchange membrane
REN	Region of electrical neutrality
SCR	Space charge region
wQEL	Without Quasi-equilibrium boundary layer
QCD	Quasi-uniform charge distribution

## List of Symbols

The following list of symbols is used in this manuscript:

$C_{0}$	The initial concentration of the solution
$C_{1}$	The concentration of cations
$C_{2}$	The concentration of anions
*H*	The distance between membranes (Channel width)
$j_{1}$	The flux of cations
$j_{2}$	The flux of anions
$D_{1}$	The duffusion of cations
$D_{2}$	The duffusion of anions
φ	The electric field potential (Potential jump)
$d_{1}$	The initial value of the potential
$d_{2}$	The speed of the potential sweep
$\varepsilon_{\alpha }$	The absolute permittivity of the solution
*F*	The Faraday constant
*R*	The gas constant
*T*	The absolute temperature
*t*	The time
$t_{n}$	The space charge breakdown start time
$\varphi_{n}$	The space charge breakdown start potential jump

## Appendix A

### Appendix A.1. Basic Mathematical Model

The basic mathematical model of one-dimensional unsteady transfer of salt ions into the section of the desalination channel [6] formed by anion-exchange and cation-exchange membranes in the potentiodynamic mode in a dimensional form is determined by the boundary value problem formulated below.

#### Appendix A.1.1. System of Equations

(A1) $$\frac{\partial C_{1}}{\partial t}=-\frac{\partial j_{1}}{\partial x}$$

(A2) $$\frac{\partial C_{2}}{\partial t}=-\frac{\partial j_{2}}{\partial x}$$

(A3) $$j_{1}=-\frac{F}{RT}D_{1}C_{1}\frac{\partial \varphi }{\partial x}-D_{1}\frac{\partial C_{1}}{\partial x}$$

(A4) $$j_{2}=\frac{F}{RT}D_{2}C_{2}\frac{\partial \varphi }{\partial x}-D_{2}\frac{\partial C_{2}}{\partial x}$$

(A5) $$\frac{\partial^{2}\varphi }{\partial x^{2}}=\frac{F}{\varepsilon_{\alpha }}\left(C_{1}-C_{2}\right)$$

where $j_{1},j_{2},C_{1},C_{2}$ are the fluxes and concentrations of cations, anions in the solution, respectively, $D_{1},D_{2}$ are the diffusion coefficients of cations and anions, φ is the electric field potential, $\varepsilon_{\alpha }$ is the absolute permittivity of the solution, *F* is the Faraday constant, *R* is the gas constant, *T* is the absolute temperature, and *t* is the time.

#### Appendix A.1.2. Boundary Conditions

At x=0:

(A6) $${\left.\left(-\frac{F}{RT}D_{1}C_{1}\frac{\partial \varphi }{\partial x}-D_{1}\frac{\partial C_{1}}{\partial x}\right)\right|}_{x=0}=0$$

(A7) $$C_{2}\left(t,0\right)=C_{2A}$$

(A8) $$\varphi \left(t,0\right)=d_{1}+d_{2}\cdot t,$$

where $d_{1}$—the initial value of the potential, $d_{2}$—the speed of the potential sweep.

At x=H:

(A9) $${\left.\left(\frac{F}{RT}D_{2}C_{2}\frac{\partial \varphi }{\partial x}-D_{2}\frac{\partial C_{2}}{\partial x}\right)\right|}_{x=0}=0$$

(A10) $$C_{1}\left(t,H\right)=C_{1K}$$

(A11) φ(t,H)=0

Initial conditions (t=0):

(A12) $$C_{1}\left(0,x\right)=C_{10}$$

(A13) $$C_{2}\left(0,x\right)=C_{20}$$

(A14) φ(0,x)=0

The boundary value problem depends on the following 7 variable input parameters: *H*, $C_{10}$, $C_{20}$, $C_{1K}$, $C_{2A}$, $d_{1}$ and $d_{2}$. To make the numerical results more visible, we set $C_{10}=C_{20}=C_{1K}=C_{2A}=C_{0}$, where $C_{0}$ is the initial concentration of the solution. It is precisely the use of the method proposed below that makes it possible not to lose the generality of the formulation.

### Appendix A.2. Characteristic Quantities and Transition to Dimensionless Form

Let us move on to dimensionless quantities using the formulas, where (*u*) is the index of the dimensionless quantity, the subscript (0) indicates the characteristic quantities:

$$x^{\left(u\right)}=\frac{x}{H},t^{\left(u\right)}=\frac{t}{t_{0}},C_{i}^{\left(u\right)}=\frac{C_{i}}{C_{0}},j_{i}^{\left(u\right)}=\frac{j_{i}}{j_{0}},D_{i}^{\left(u\right)}=\frac{D_{i}}{D_{0}}$$

$$\varphi_{i}^{\left(u\right)}=\frac{\varphi }{\varphi_{0}},\varepsilon_{i}^{\left(u\right)}=\frac{\varepsilon_{\alpha }}{b_{0}},I^{\left(u\right)}=\frac{I}{I_{0}},d_{1}^{\left(u\right)}=\frac{d_{1}}{\varphi_{0}},d_{2}^{\left(u\right)}=\frac{d_{2}}{d_{0}}$$

#### Appendix A.2.1. Characteristic Quantities, Their Physical Meaning

Two characteristic quantities are obvious. This is the initial concentration of the solution $C_{0}$, which really varies from $10^{-2}$ mol/m${}^{3}$ to $10^{2}$ mol/m${}^{3}$ and the channel width H=1 mm, the remaining characteristic values must be chosen so that the boundary value problem has the simplest form. Let us take as $D_{0}=\frac{2D_{1}D_{2}}{z_{1}D_{1}-z_{2}D_{2}}$ is the diffusion coefficient of the electrolyte, $\varphi_{0}$ is the thermal potential [28], $I_{0}=\frac{2FD_{0}C_{0}}{H}$ is the limiting diffusion current, $j_{0}=\frac{2D_{0}C_{0}}{H}$ is the ion flux corresponding to the limiting current, $t_{0}=\frac{C_{0}H}{j_{0}}$ is the time of diffusion of ions through the channel cross section, $d_{0}=\frac{\varphi_{0}}{t_{0}}=\frac{\varphi_{0}D_{0}}{H^{2}}$ is the characteristic value of the potential sweep rate, and $b_{0}=\frac{H^{2}FC_{0}}{\varphi_{0}}$ is a characteristic value having the dimension of an electric constant.

The physical meaning of the quantity $b_{0}$ can be seen, for example, by rewriting the formula $\frac{\varepsilon_{\alpha }}{b_{0}}$ in the form $\frac{\varepsilon_{\alpha }}{b_{0}}=\frac{\varepsilon_{\alpha }S}{H}:\frac{b_{0}S}{H}=\frac{C_{k}}{C_{T}}$, where $C_{k}$ is the capacitance of a flat capacitor with the area of the plates *S* and the thickness of the dielectric *H*, and $C_{T}=\frac{b_{0}S}{H}=\frac{HFC_{0}S}{\varphi_{0}}$ is the capacitance a channel for desalination of an electrolyte solution with a width of *H*, with an area of membranes *S* and with a concentration of $C_{0}$, considered as an ionistor (supercapacitor). Another physical interpretation of the small parameter $\varepsilon^{\left(u\right)}$ is defined in [29] as the square of the ratio of the Debye length to the characteristic linear size, in this case, to the channel width.

#### Appendix A.2.2. Estimation of Characteristic Quantities

$$\begin{array}{cc} \; & \varphi_{0}=\frac{RT}{F}=0.02566\left[V\right],\\ \; & b_{0}:3.8594\times 10^{-2}\left[\frac{Kl}{\mathrm{mV}}\right]\leq b_{0}\leq 3.8594\times 10^{2}\left[\frac{Kl}{\mathrm{mV}}\right]. \end{array}$$

The value $D_{0}$, and therefore $I_{0},j_{0},t_{0}$ depend on the salt. For example, for a KCl solution, we have:

$$\begin{array}{cc} \; & D_{0}=\frac{2D_{1}D_{2}}{z_{1}D_{1}-z_{2}D_{2}}\approx 2.003\times 10^{-9}\left[\frac{m^{2}}{s}\right],d_{0}=5\cdot 10^{-5}\left[V/s\right],4.9\leq t_{0}\leq 4.9\times 10^{3}\left[s\right],\\ \; & 4.007\times 10^{-8}\left[\frac{\mathrm{mol}}{m^{2}s}\right]\leq j_{0}\leq 4.007\times 10^{-4}\left[\frac{\mathrm{mol}}{m^{2}s}\right],\\ \; & 0.3865\times 10^{-2}\left[A/m^{2}\right]\leq I_{0}\leq 0.3865\times 10^{2}\left[A/m^{2}\right]. \end{array}$$

#### Appendix A.2.3. Trivial Similarity Criteria, Their Physical Meaning and Estimation of Values

The boundary value problem depends only on three dimensionless parameters $\varepsilon^{\left(u\right)}$, $d_{1}^{\left(u\right)}$, $d_{2}^{\left(u\right)}$. Let us estimate their values: $1.859\times 10^{-12}\leq \varepsilon^{\left(u\right)}\leq 1.859\times 10^{-8}$, $5\leq d_{2}^{\left(u\right)}\leq 5\times 10^{3}\left[V\right]$.

**Table A1:** Specific values of trivial similarity criteria from $C_{0}$. The last line shows the order of the dimensionless thickness of the quasi-equilibrium region of the space charge at a dimensionless width of the desalination channel equal to 1.

$C_{0},$ mol/m${}^{3}$	0.01	0.1	1	10	100
$I_{0}=I_{lim},A/m^{2}$	0.003865	0.03865	0.3865	3.865	38.65
$\varepsilon^{\left(u\right)}$	$1.859\times 10^{-8}$	$1.859\times 10^{-9}$	$1.859\times 10^{-10}$	$1.859\times 10^{-11}$	$1.859\times 10^{-12}$
$\sqrt{\varepsilon^{\left(u\right)}}\left|ln\varepsilon^{\left(u\right)}\right|$	$2.426\times 10^{-3}$	$8.6\times 10^{-4}$	$3.0\times 10^{-4}$	$1.0\times 10^{-4}$	$3.7\times 10^{-5}$

### Appendix A.3. Basic Mathematical Model in Dimensionless Form

#### Appendix A.3.1. System of Equations in Dimensionless Form

Taking into account non-trivial similarity criteria, the dimensionless boundary value problem can be written in the form (the index “*u*” is omitted for simplicity):

(A15) $$\frac{\partial C_{i}}{\partial t}=-\frac{\partial j_{i}}{\partial x},i=1,2$$

(A16) $$j_{i}=-z_{i}D_{i}C_{i}\frac{\partial \varphi }{\partial x}-D_{i}\frac{\partial C_{i}}{\partial x},i=1,2$$

(A17) $$\varepsilon \frac{\partial^{2}\varphi }{\partial x^{2}}=-\left(C_{1}-C_{2}\right)$$

#### Appendix A.3.2. Boundary Conditions

At x=0:

(A18) $${\left.\left(-C_{1}\frac{\partial \varphi }{\partial x}-\frac{\partial C_{1}}{\partial x}\right)\right|}_{x=0}=0$$

(A19) $$C_{2}\left(t,0\right)=C_{2A}=1$$

(A20) $$\varphi \left(t,0\right)=d_{1}+d_{2}\times t,$$

At x=1:

(A21) $${\left.\left(C_{2}\frac{\partial \varphi }{\partial x}-\frac{\partial C_{2}}{\partial x}\right)\right|}_{x=0}=0$$

(A22) $$C_{1}\left(t,1\right)=C_{1K}=1$$

(A23) φ(t,1)=0

Initial conditions (t=0):

(A24) $$C_{i}\left(0,x\right)=C_{i}\left(x\right)=1,i=1,2,\varphi \left(0,x\right)=0$$

As can be seen from the system of equations and boundary conditions, there are only three parameters: ε, $d_{1}$ and $d_{2}$.
